# PMAIP1 Enhances DNA Damage and Induces ROS‐Mediated Mitochondrial Dysfunction to Suppress Tumorigenesis in Triple‐Negative Breast Cancer

**DOI:** 10.1155/tbj/7056712

**Published:** 2025-12-30

**Authors:** Fangjian Shang, Lei Xu, Hongzhi Liu, Xin Dong, Huangfei Wu, Liping Yin, Lijuan Yan, Yixin Qi, Liyan Zhao

**Affiliations:** ^1^ Department of General Surgery, The First Hospital of Hebei Medical University, Shijiazhuang, 050023, Hebei, China, hebmu.edu.cn; ^2^ School of Basic Medical Sciences, First Affiliated Hospital of Xingtai Medical College, Xingtai, 054099, Hebei, China; ^3^ Department of Gland Surgery, Hebei General Hospital, Shijiazhuang, 050051, Hebei, China, hebmu.edu.cn; ^4^ School of Basic Medical Sciences, Xingtai Medical College, Xingtai, 054000, Hebei, China; ^5^ Breast Center, The Fourth Hospital of Hebei Medical University, Shijiazhuang, 050011, Hebei, China, hebmu.edu.cn

**Keywords:** apoptosis, DNA damage, mitochondrial dysfunction, PMAIP1 (NOXA), triple-negative breast cancer (TNBC)

## Abstract

**Background:**

PMAIP1 (NOXA) is a pro‐apoptotic factor that is closely related to cancer development, but its role in triple‐negative breast cancer (TNBC) is unclear. This study aimed to explore the effect of PMAIP1 on TNBC cell viability, apoptosis, DNA damage, and mitochondrial function.

**Methods:**

qRT‐PCR and western blot were used to detect the expression level of PMAIP1 in TNBC tissues and cells, and its biological role was evaluated in combination with MTT, TUNEL, comet assay, and mitochondrial function indicators (ROS, ATP, mtDNA, and JC‐1).

**Results:**

PMAIP1 is significantly upregulated in TNBC and is negatively correlated with cell viability: Overexpression of PMAIP1 inhibits cell viability, while knockdown of PMAIP1 enhances viability. Upregulation of PMAIP1 promotes apoptosis by increasing the Bax/Bcl‐2 ratio, induces DNA damage, elevates ROS levels, and reduces ATP, mtDNA, and JC‐1 levels, leading to mitochondrial dysfunction; conversely, knockdown of PMAIP1 alleviates these changes.

**Conclusion:**

PMAIP1 exerts a tumor suppressor effect by regulating apoptosis, DNA damage, and mitochondrial dysfunction, providing potential target support for the treatment of TNBC.

## 1. Introduction

Triple‐negative breast cancer (TNBC), characterized by lack of estrogen receptor (ER), progesterone receptor (PR) and human epidermal growth factor receptor 2 (HER2) expression, accounts for 15%–20% of all breast cancer cases and is associated with poor prognosis, high recurrence rate, and limited treatment options [1–4]. Unlike other breast cancer subtypes, TNBC lacks well‐established molecular targets, highlighting the urgent need to explore new regulatory pathways and potential therapeutic targets [5, 6].

Apoptosis is a fundamental cellular process that is often dysregulated in TNBC, contributing to tumor initiation and progression [7]. BCL‐2 family proteins, including anti‐apoptotic members (such as BCL2 and BCL2L1) and pro‐apoptotic members (such as Bax and PMAIP1), play key roles in regulating mitochondria‐mediated apoptosis [8, 9]. PMAIP1, also known as NOXA, is a pro‐apoptotic member of the Bcl‐2 protein family and is involved in the regulation of mitochondria‐mediated apoptosis, DNA damage repair, and oxidative stress responses in various cancers [10, 11]. DNA damage is another hallmark of cancer cells, often exacerbated by oxidative stress and defects in DNA repair mechanisms [12, 13]. New research shows that PMAIP1 plays a key role in regulating DNA damage markers such as γH2AX and p‐ATM, determining its involvement in the activation of DNA damage signaling cascades [14].

Mitochondrial dysfunction and reactive oxygen species (ROS) accumulation are also key factors in the pathogenesis of TNBC. Elevated ROS levels can induce mitochondrial membrane depolarization, reduce ATP production, and damage mitochondrial DNA (mtDNA), ultimately leading to cell death [15, 16]. Studies have shown that PMAIP1 can amplify ROS production and mitochondrial dysfunction in cancer cells, making it a promising target for redox‐based cancer therapy [17]. Furthermore, interactions between PMAIP1 and mitochondrial dynamics, including changes in mitochondrial membrane potential and mtDNA integrity, are associated with cancer cell apoptosis [11, 18].

Although increasing evidence suggests that PMAIP1 is associated with apoptosis, DNA damage, and mitochondrial dysfunction in various cancers, its specific role in TNBC remains unclear. Therefore, in this study, we focused on elucidating the functional role of PMAIP1 in TNBC, particularly its involvement in regulating cell viability, apoptosis, DNA damage, and mitochondrial dysfunction. Understanding the molecular mechanisms by which PMAIP1 affects these pathways could provide valuable insights into TNBC biology and provide new therapeutic strategies for TNBC.

Building on our previous work showing that ATG5 inhibition stabilizes PMAIP1 to promote apoptosis in TNBC [19], this study further investigates the downstream mechanisms of PMAIP1. We focused on ROS‐mediated mitochondrial dysfunction and DNA damage to clarify the redox‐dependent pathway linking PMAIP1 to TNBC cell apoptosis and to provide new evidence for its therapeutic potential.

## 2. Materials and Methods

### 2.1. Data Collection and Analysis From Public Databases

Transcriptomic data and corresponding clinical information for TNBC were obtained from two independent cohorts: The Cancer Genome Atlas (TCGA) database (https://tcga-data.nci.nih.gov/) and the Molecular Taxonomy of Breast Cancer International Consortium (METABRIC) dataset (https://www.cbioportal.org/). For the TCGA cohort, a total of 1217 breast cancer samples were initially included, and TNBC cases were identified by PAM50 molecular subtyping, resulting in a final dataset of 114 samples. For the METABRIC cohort, TNBC cases (*n* = 320) and their corresponding clinical data were extracted from cBioPortal.

Principal component analysis (PCA) was conducted to assess the global transcriptional differences between TNBC and normal breast tissues. PMAIP1 expression levels were compared between TNBC and normal tissues in both TCGA and METABRIC datasets. In the METABRIC TNBC subset, patients were stratified into high‐ and low‐expression groups based on the median PMAIP1 expression level, and survival analysis for overall survival was performed using the Kaplan–Meier method with log‐rank testing.

Differentially expressed genes (DEGs) between high and low PMAIP1 groups were identified using the DESeq2 package in R (Version 4.3.0), with thresholds set at |log_2_ fold change| ≥ 1 and adjusted *p* value < 0.05. DEG expression patterns were visualized using hierarchical clustering heatmaps. Functional enrichment analyses, including Gene Ontology (GO) classification (biological processes [BP], cellular components [CCs], and molecular functions [MFs]) and Kyoto Encyclopedia of Genes and Genomes (KEGG) pathway analysis, were conducted using the ClusterProfiler package. Finally, protein–protein interaction (PPI) analysis for PMAIP1‐associated genes was constructed using the STRING database (https://string-db.org/) and visualized using Cytoscape software.

### 2.2. Cell Culture and Grouping

The human normal mammary epithelial cell line MCF10A (CRL‐10317) and the TNBC cell lines MDA‐MB‐468 (HTB‐132) and MDA‐MB‐231 (HTB‐26) were obtained from the American Type Culture Collection (ATCC). All cell lines were cultured in Dulbecco’s Modified Eagle Medium (DMEM, Gibco, USA) supplemented with 10% fetal bovine serum (FBS) and 1% penicillin–streptomycin and maintained in a 37°C incubator with 5% CO_2_. In this experiment, MDA‐MB‐468 cells were divided into three groups: control group (cultured under standard conditions without any treatment), vector group (transfected with the negative pcDNA3.1 vector as a control), and oe‐PMAIP1 group (transfected with the pcDNA3.1‐PMAIP1 plasmid to overexpress PMAIP1). MDA‐MB‐231 cells were also divided into three groups: the control group (cultured under standard conditions without any treatment), the sh‐NC group (transfected with the negative shRNA vector as a control), and the sh‐PMAIP1 group (transfected with PMAIP1‐specific shRNA to knock down PMAIP1 expression).

### 2.3. Kit Detection

The levels of ROS, ATP, mtDNA, and mitochondrial membrane potential (JC‐1) in MDA‐MB‐468 and MDA‐MB‐231 cells were assessed using commercial assay kits. Briefly, after treatment, cell samples were collected and processed according to the manufacturer’s instructions. ROS levels were measured using the DCFH‐DA probe (Sevier, China). The cells were then analyzed for intracellular ROS levels using a fluorescence microplate reader (Thermo Fisher Scientific, USA). ATP content was measured using an ATP detection kit (Beyotime, China), and the fluorescence or optical density of the cell lysates was determined via colorimetric analysis. mtDNA levels were assessed by PCR amplification of specific mtDNA regions and comparing the results to nuclear DNA, calculating the ratio of mtDNA to nuclear DNA. Mitochondrial membrane potential was evaluated using the JC‐1 staining method, where JC‐1 dye aggregates to form red fluorescence in healthy mitochondria and produces green fluorescence in depolarized mitochondria. The ratio of red to green fluorescence was analyzed using a fluorescence microscope (Olympus, Japan) to assess changes in mitochondrial membrane potential.

### 2.4. TUNEL

Apoptosis detection was performed using a TUNEL kit (Beyotime, China). First, the treated cells were fixed in 4% paraformaldehyde solution at room temperature for 30 min. After fixation, cells were washed three times (5 min each time) with PBS (ThermoFisher Scientific, USA). The cells were then permeabilized with 0.3% Triton X‐100 solution for 5 min to enhance the permeability of the cell membrane and washed three times with PBS. Then refer to the TUNEL kit instructions, add an appropriate amount of TUNEL reaction solution, and incubate the cells at 37°C in the dark for 1 h. After incubation, wash cells three times with PBS (5 min each time). Finally, the fluorescent images of TUNEL‐positive cells were observed under a fluorescence microscope (Olympus, Japan) and photographed, and the proportion of apoptotic cells was calculated as the apoptosis rate.

### 2.5. Comet Assay

A comet electrophoresis kit (Beyotime, China) was used to detect DNA damage. Treated cells were collected and prepared into a single‐cell suspension, which was then embedded in low‐melting‐point agarose and placed onto pretreated glass slides. A coverslip was placed on top, and the slides were left at 4°C for 10 min to solidify. Next, the slides were placed in lysis buffer and lysed for 2 h at 4°C in the dark. After lysis, perform electrophoresis (25 V, 300 mA) in alkaline electrophoresis buffer for 20 min. After electrophoresis, the slides were neutralized with neutral buffer and washed twice with distilled water. Fluorescent staining was performed after washing. Finally, the migration of DNA was observed by fluorescence microscopy (Olympus, Japan); the comet tail DNA% (comet tail DNA level) and tail moment (comet tail distance) were recorded; and the DNA damage level of the cells was evaluated using ImageJ software based on these parameters.

### 2.6. Western Blotting Assay

Western blot was used to detect the expression levels of related proteins. Total protein was extracted with RIPA lysis buffer containing protease inhibitors, and the protein concentration was detected using a BCA protein assay kit (Beyotime, China). A total of 30 μg/well protein lysate was separated by 10% SDS–PAGE gel electrophoresis and transferred to a PVDF membrane (Millipore, USA). After blocking in 5% skim milk for 2 h at room temperature, the membrane was incubated with primary antibodies overnight and then with secondary antibodies conjugated to anti‐IgG HRP for 2 h. Finally, the protein bands were visualized using enhanced chemiluminescence (ECL) reagent (Thermo Fisher, MA, USA). GAPDH (ab9485) was used as the internal control, and ImageJ software was used to quantitatively analyze the density of the protein bands. Primary antibodies, including PMAIP1 (ab13654), Bax (ab182733), Bcl‐2 (ab182858), γH2AX (ab81299), p‐ATM (Ser1981) (ab36810), and p53 (ab26) were purchased from Abcam company.

### 2.7. qRT‐PCR

Total RNA was extracted from tissue samples and cultured cells using TRIzol reagent (Invitrogen, Carlsbad, USA). Complementary DNA (cDNA) was synthesized according to the instructions of the PrimeScript RT reagent kit (Takara, Japan). Quantitative reverse transcription polymerase chain reaction (qRT‐PCR) was then performed using the qRT‐PCR kit (Takara, Japan). The primer sequences of this study are as follows: PMAIP1 forward primer 5′‐GAG​GAA​CAA​GTG​CAA​GTA​GCT​G‐3′, reverse primer 5′‐AGG​TTC​CTG​AGC​AGA​AGA​GTT‐3′. Using GAPDH as the internal reference, the relative expression of PMAIP1 in different samples was calculated according to the 2^−ΔΔCt^ method.

### 2.8. MTT Assay

Cell viability under different treatment conditions (0 h, 12 h, 24 h, 48 h, and 72 h) was assessed using the MTT assay. Briefly, 5 × 10^3^ cells of each type were seeded into 96‐well plates and incubated for 24 h at 37°C with 5% CO_2_ to allow cell attachment. Subsequently, 100 μL of MTT solution (5 mg/mL) was added to each well, and cells were incubated for an additional 4 h, allowing the MTT to be reduced to purple formazan within the cells. The MTT solution was then removed, and 150 μL of dimethyl sulfoxide (DMSO) was added to dissolve the formazan. The plates were gently shaken until the formazan was completely dissolved. Finally, the absorbance (OD) at 570 nm was measured using a microplate reader (PerkinElmer, MA, USA) to evaluate cell proliferation activity.

### 2.9. Statistical Analysis

All experimental data were analyzed using GraphPad statistical software (GraphPad Prism 8.0). Comparisons between two groups were performed using Student’s *t*‐test, and comparisons between multiple groups were performed using one‐way ANOVA followed by Tukey’s post hoc test. Data results were expressed as the mean ± standard deviation (SD) of at least three independent experiments, and *p* < 0.05 indicates statistical significance.

## 3. Results

### 3.1. PMAIP1 Is Highly Expressed and Clinically Relevant in TNBC

To explore the biological significance of PMAIP1 in TNBC, we first examined its expression pattern and prognostic relevance using public datasets and cell models. PCA revealed distinct clustering between normal breast tissues from GTEx and TNBC samples from the METABRIC dataset, indicating clear transcriptomic differences between the two groups (Figure 1(a)). Consistently, database‐based expression comparisons demonstrated that PMAIP1 expression was significantly higher in TNBC tissues compared with normal controls in both the METABRIC (Figure 1(b)) and TCGA datasets (Figure 1(c)). In the METABRIC TNBC subset, Kaplan–Meier survival analysis revealed that high PMAIP1 expression was significantly associated with shorter overall survival (log‐rank *p* = 0.0081), suggesting a potential role as a negative prognostic biomarker (Figure 1(d)).

Figure 1Data analysis of PMAIP1 in TNBC. (a) Principal component analysis (PCA) showing clear separation between normal breast tissues from the GTEx database and TNBC samples from the METABRIC dataset. (b) Expression levels of PMAIP1 in TNBC and normal tissues in the METABRIC cohort (*n* = 320). (c) Expression levels of PMAIP1 in TNBC and normal tissues in the TCGA cohort (*n* = 114). (d) Kaplan–Meier survival curves for overall survival in the METABRIC TNBC subset, stratified by PMAIP1 expression level (high vs. low). (e) Volcano plot displaying differentially expressed genes (DEGs) between high and low PMAIP1 expression groups in the METABRIC TNBC subset. (f) Heatmap showing the expression patterns of DEGs in PMAIP1 high‐ and low‐expression groups. (g) GO enrichment analysis of DEGs. (h) GO enrichment analysis of PMAIP1‐related genes in the TCGA TNBC dataset. (i) KEGG pathway enrichment analysis of PMAIP1‐related genes. (j) Protein–protein interaction (PPI) network of PMAIP1‐associated genes constructed using the STRING database. Data are represented as mean ± SD. Statistical significance was determined using Student’s *t*‐test; ^∗∗∗^ *p* < 0.001 and ^∗∗∗∗^ *p* < 0.0001.

**Figure:**
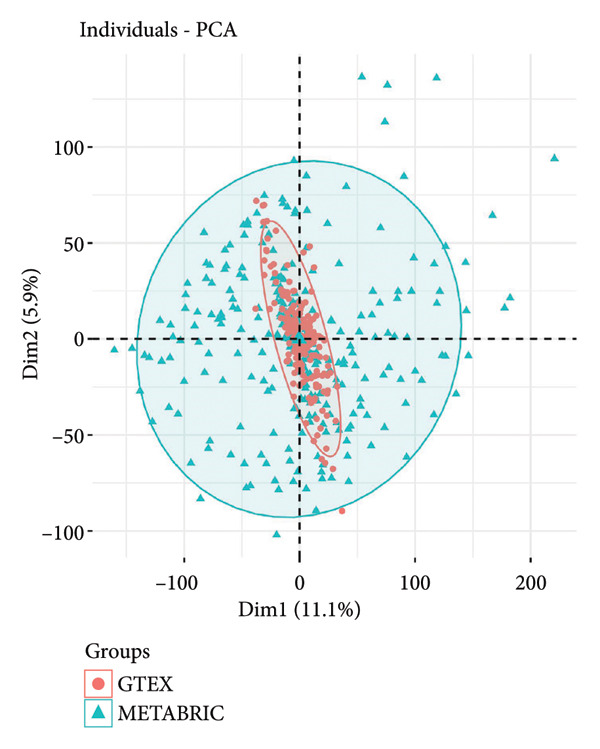
(a)

**Figure:**
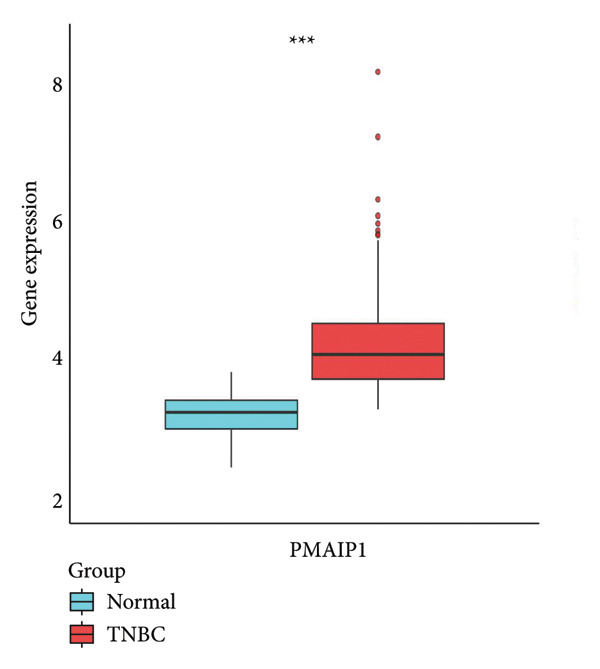
(b)

**Figure:**
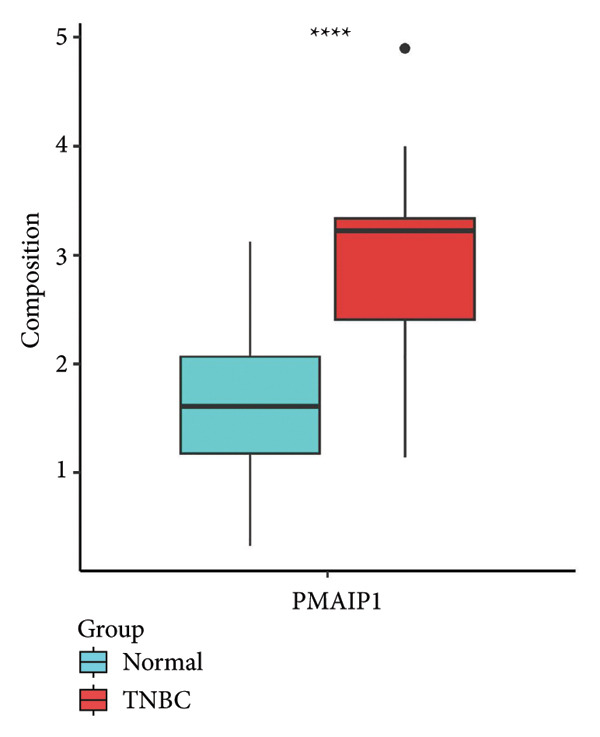
(c)

**Figure:**
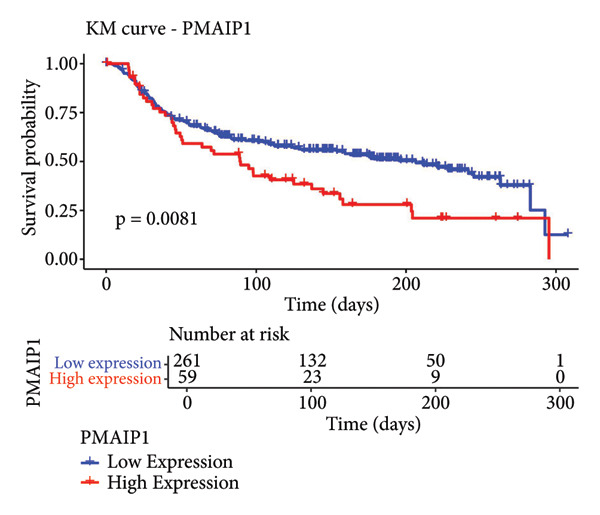
(d)

**Figure:**
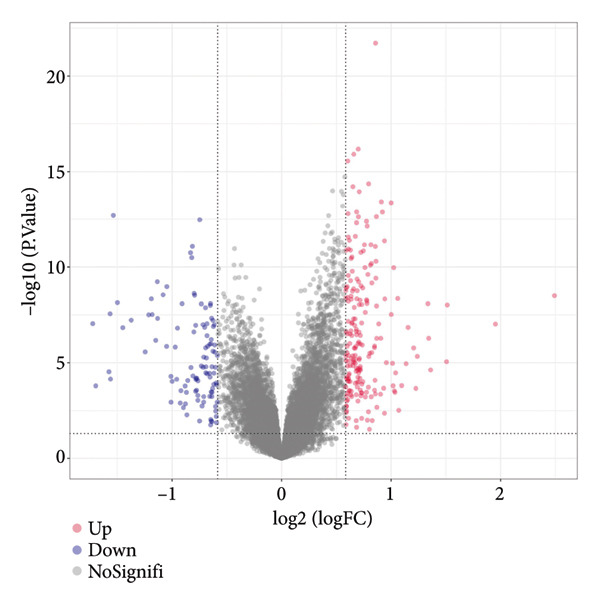
(e)

**Figure:**
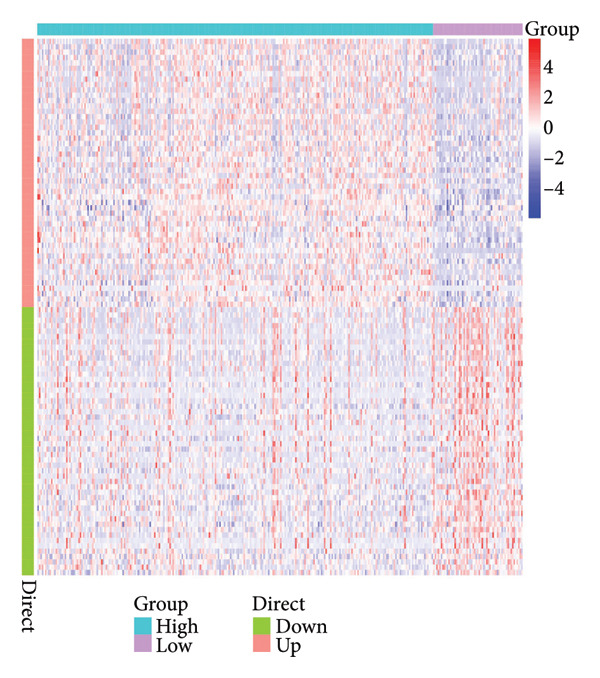
(f)

**Figure:**
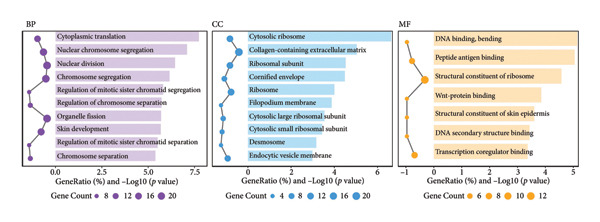
(g)

**Figure:**
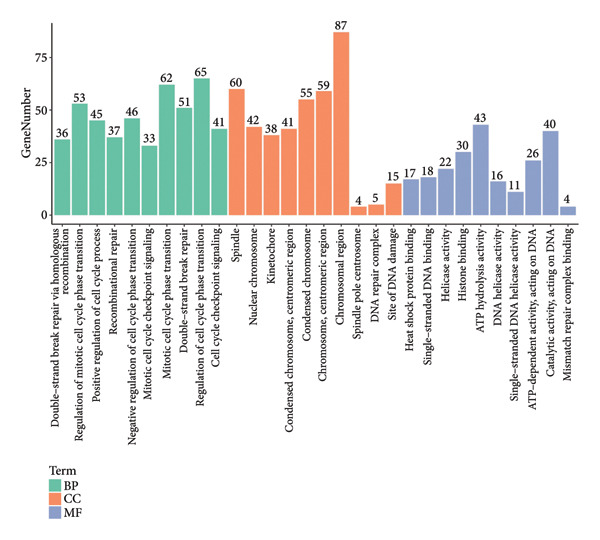
(h)

**Figure:**
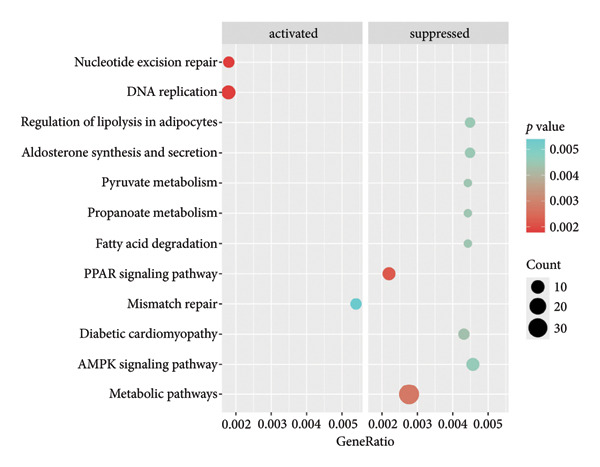
(i)

**Figure:**
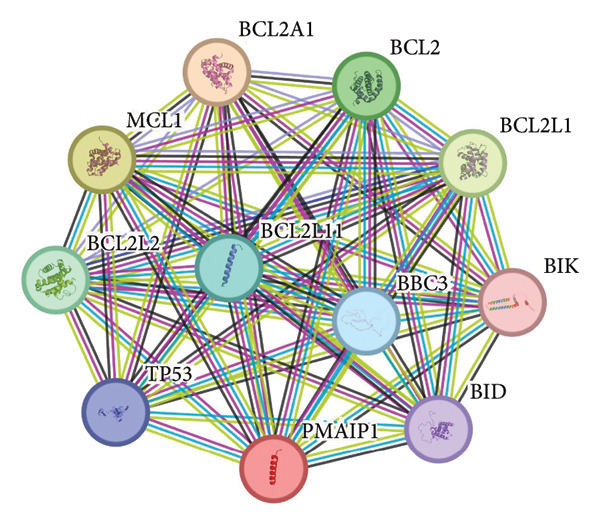
(j)

Differential expression analysis between PMAIP1 high‐ and low‐expression TNBC groups identified a distinct set of upregulated and downregulated genes (Figure 1(e)), and hierarchical clustering heatmaps showed clear segregation between the two groups (Figure 1(f)). GO enrichment analysis indicated that these genes were predominantly involved in BP such as chromosome segregation, nuclear division, and regulation of sister chromatid separation; CCs such as the cytosolic ribosome and extracellular matrix; and MFs such as DNA binding and ribosomal structural constituents (Figures 1(g) and 1(h)). KEGG pathway enrichment analysis further revealed significant enrichment in DNA repair, oxidative phosphorylation, and metabolic pathways (Figure 1(i)). PPI network analysis highlighted close interactions of PMAIP1 with apoptosis‐ and survival‐related proteins, particularly members of the BCL2 family (BCL2, BCL2L1, MCL1) and TP53 (Figure 1(j)).

Together, these findings demonstrated that PMAIP1 was consistently upregulated in TNBC across datasets and was associated with poorer patient survival, linking it to gene networks involved in apoptosis, DNA damage repair, and oxidative metabolism. Given this enrichment in oxidative stress‐related pathways, we next investigated whether PMAIP1 modulates mitochondrial function and redox homeostasis in TNBC cells.

### 3.2. PMAIP1 Overexpression Disrupts Mitochondrial Function and Elevates ROS Levels

As mitochondrial dysfunction is a major source of cellular ROS, we assessed the impact of PMAIP1 on mitochondrial homeostasis in TNBC cells. As shown in Figure 2, PMAIP1 overexpression markedly elevated intracellular ROS levels, accompanied by a notable reduction in ATP production, mtDNA content, and mitochondrial membrane potential (JC‐1 red/green fluorescence ratio). Conversely, PMAIP1 knockdown reduced intracellular ROS accumulation and restored mitochondrial function, as evidenced by increased ATP, mtDNA, and JC‐1 levels. These findings suggest that PMAIP1 overexpression disrupts mitochondrial integrity through ROS accumulation, whereas its knockdown exerts a protective effect by preserving mitochondrial homeostasis. Collectively, these data indicate that PMAIP1 triggers mitochondrial dysfunction accompanied by ROS accumulation. To elucidate the downstream consequences of this oxidative stress, we next examined whether PMAIP1‐induced ROS elevation promotes apoptosis and DNA damage.

Figure 2Effects of different treatments on ROS levels, mitochondrial function and membrane potential in MDA‐MB‐468 and MDA‐MB‐231 cells. (a) ROS levels of MDA‐MB‐468 and MDA‐MB‐231 cells in different treatment groups. Scale bar: 50 μm. (b and c) ATP and mitochondrial DNA (mtDNA) levels of MDA‐MB‐468 and MDA‐MB‐231 cells in different treatment groups. (d) Levels of mitochondrial membrane potential (JC‐1) in MDA‐MB‐468 and MDA‐MB‐231 cells in different treatment groups. Scale bar: 50 μm. Data are represented as mean ± SD (*n* = 3). Statistical significance was assessed using one‐way ANOVA with Tukey’s post hoc test; ^∗^ *p* < 0.05 and ^∗∗^ *p* < 0.01.

**Figure:**
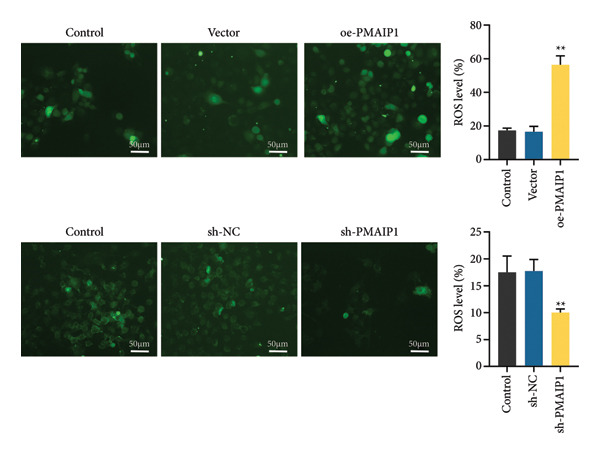
(a)

**Figure:**
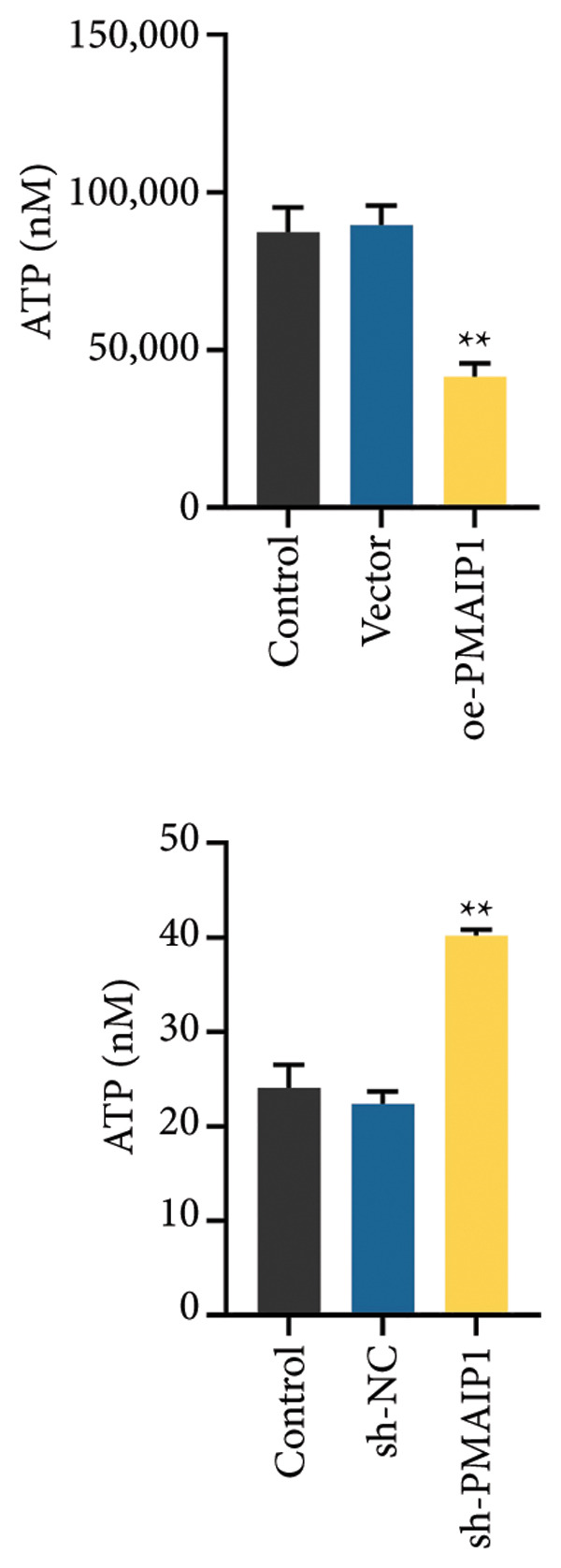
(b)

**Figure:**
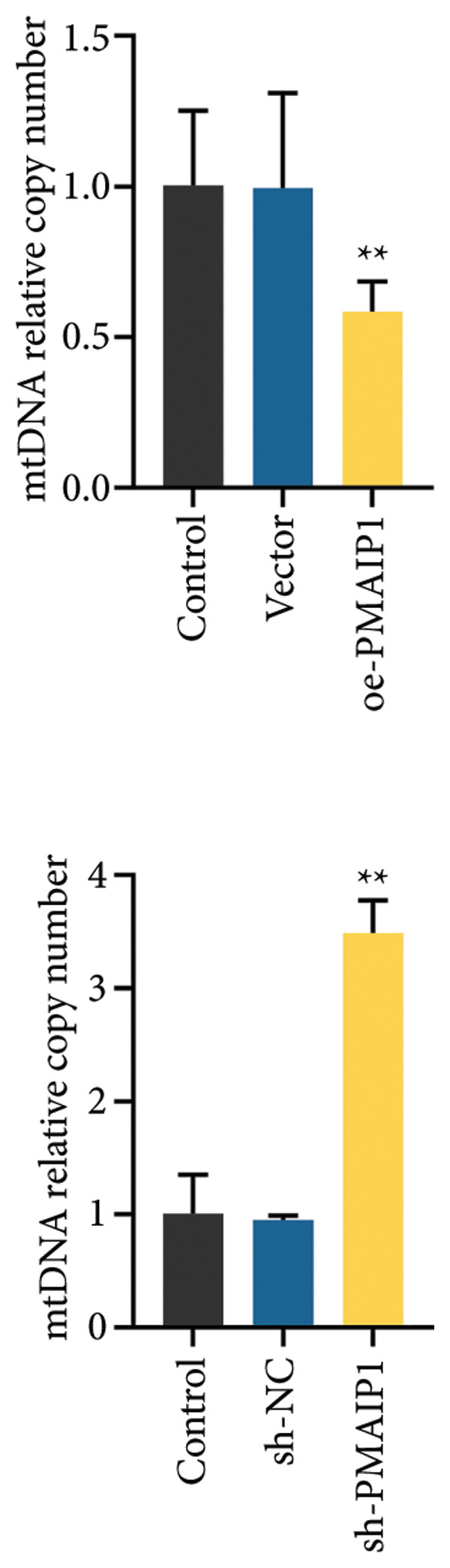
(c)

**Figure:**
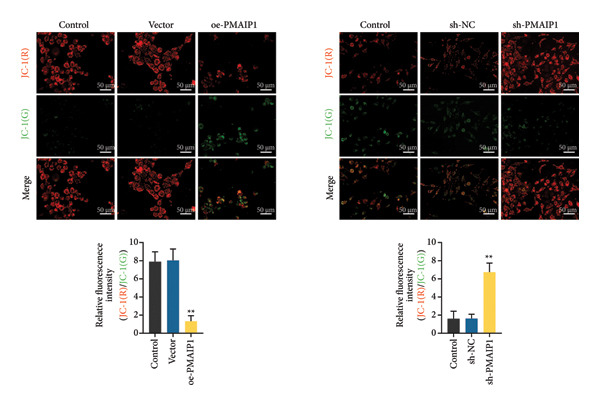
(d)

### 3.3. ROS‐Dependent Mitochondrial Dysfunction Induces Apoptosis and DNA Damage in TNBC Cells

To explore how PMAIP1‐driven oxidative stress influences cell fate and genomic stability, apoptosis and DNA damage were evaluated in TNBC cells. The TUNEL assay (Figure 3(a)) revealed a marked increase in apoptotic cells following PMAIP1 overexpression in MDA‐MB‐468 cells, whereas no significant difference was observed between the control and vector groups. Conversely, PMAIP1 knockdown significantly reduced apoptosis in MDA‐MB‐231 cells compared with sh‐NC controls. Western blot analysis (Figure 3(b)) further confirmed that PMAIP1 overexpression elevated the level of the pro‐apoptotic protein Bax while reducing the anti‐apoptotic protein Bcl‐2, thereby increasing the Bax/Bcl‐2 ratio. In contrast, silencing PMAIP1 decreased Bax expression and upregulated Bcl‐2.

Figure 3Effects of different treatments on apoptosis and apoptosis‐related proteins in MDA‐MB‐468 and MDA‐MB‐231 cells. (a) TUNEL detects the apoptosis levels of MDA‐MB‐468 and MDA‐MB‐231 cells in different treatment groups. (b) Western blot detects the protein expression levels of Bax and Bcl‐2 in MDA‐MB‐468 and MDA‐MB‐231 cells in different treatment groups. Data are represented as mean ± SD (*n* = 3). Statistical analysis was conducted using one‐way ANOVA with Tukey’s post hoc test; ^∗^ *p* < 0.05 and ^∗∗^ *p* < 0.01.

**Figure:**
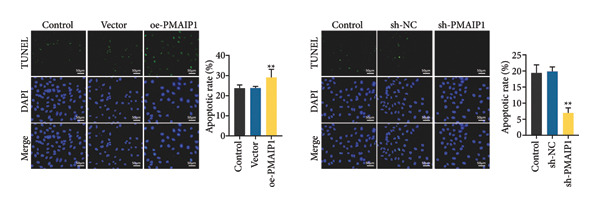
(a)

**Figure:**
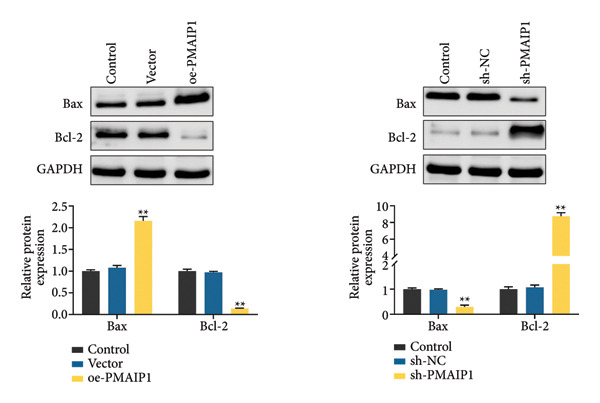
(b)

Consistent with its pro‐apoptotic role, comet assay results (Figure 4(a)) showed that PMAIP1 overexpression markedly increased DNA damage, as indicated by higher tail DNA% and tail moment values, whereas PMAIP1 knockdown produced the opposite effect. Western blot analysis (Figure 4(b)) also revealed that γH2AX, p‐ATM (Ser1981), and p53 were significantly upregulated in PMAIP1‐overexpressing MDA‐MB‐468 cells but were reduced following PMAIP1 silencing in MDA‐MB‐231 cells.

Figure 4Effects of different treatments on DNA damage and related protein expression in MDA‐MB‐468 and MDA‐MB‐231 cells. (a) Comet assay shows DNA damage (tail DNA% and tail moment) of MDA‐MB‐468 and MDA‐MB‐231 cells in different treatment groups. Scale bar: 50 μm. (b) Western blot analysis of γH2AX, p‐ATM (Ser1981), and p53 protein expression in different treatment groups. Data are represented as mean ± SD (*n* = 3). Statistical comparisons were made using one‐way ANOVA with Tukey’s post hoc test; ^∗^ *p* < 0.05 and ^∗∗^ *p* < 0.01.

**Figure:**
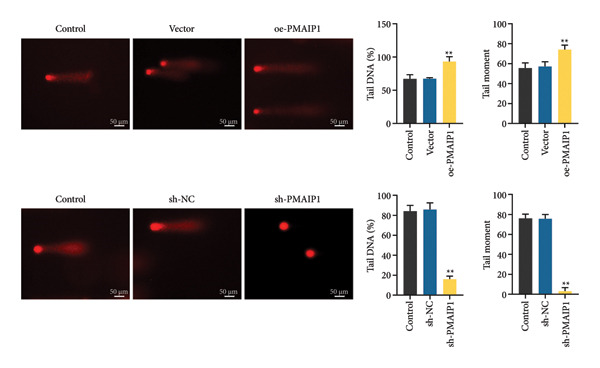
(a)

**Figure:**
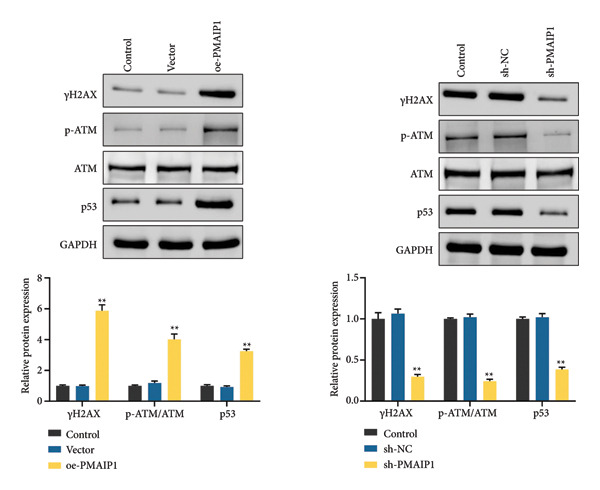
(b)

These findings suggest that PMAIP1 promotes apoptosis and DNA damage primarily through ROS‐dependent mitochondrial dysfunction, linking oxidative stress to both cell death and genomic instability in TNBC.

### 3.4. PMAIP1 Modulates TNBC Cell Viability via the ROS–Mitochondrial Pathway

To validate the functional significance of the ROS‐mitochondrial axis, we analyzed the effects of PMAIP1 on cell proliferation and viability. As shown in Figures 5(a) and 5(b), PMAIP1 mRNA and protein levels were significantly higher in MDA‐MB‐231 cells than in normal breast epithelial cells (MCF10A), whereas only a slight, non‐significant increase was observed in MDA‐MB‐468 cells. This difference likely reflects the molecular heterogeneity of TNBC subtypes. Accordingly, MDA‐MB‐468 (low baseline PMAIP1) was used for overexpression and MDA‐MB‐231 (high baseline PMAIP1) for knockdown experiments.

Figure 5The expression level of PMAIP1 in different treatment groups and its effect on the viability of MDA‐MB‐468 and MDA‐MB‐231 cells. (a and b) qRT‐PCR and western blot detected the expression level of PMAIP1 in cells of the MCF10A group, MDA‐MB‐468 group, and MDA‐MB‐231 group. (c and d) qRT‐PCR and western blot detected the expression levels of PMAIP1 in MDA‐MB‐468 and MDA‐MB‐231 cells in different treatment groups. (e) MTT detection of the viability of MDA‐MB‐468 and MDA‐MB‐231 cells at 0 h (before transfection) and 12 h, 24 h, 48 h, and 72 h after transfection with control, vector, oe‐PMAIP1, sh‐NC, or sh‐PMAIP1 constructs. Data are represented as mean ± SD (*n* = 3). Statistical comparisons were performed using one‐way ANOVA with Tukey’s post hoc test; ^∗^ *p* < 0.05 and ^∗∗^ *p* < 0.01.

**Figure:**
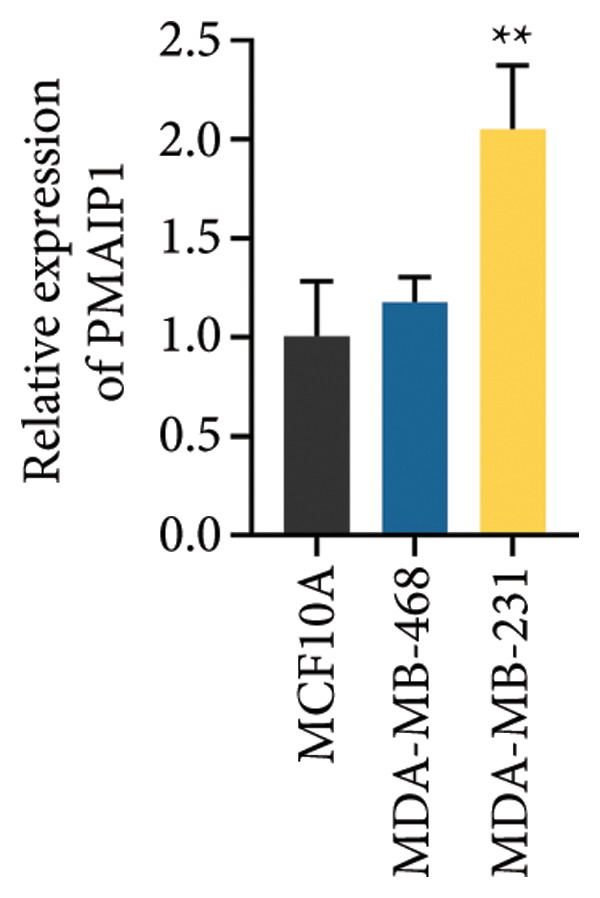
(a)

**Figure:**
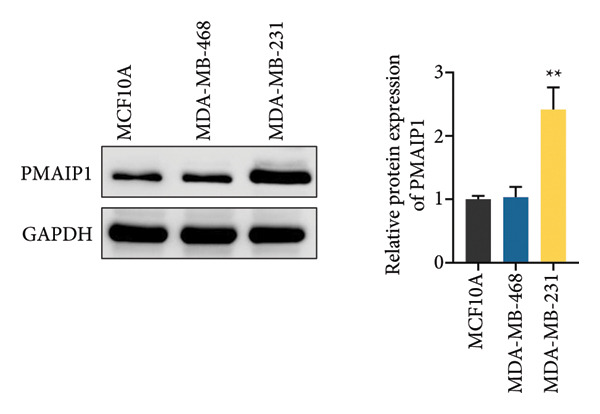
(b)

**Figure:**
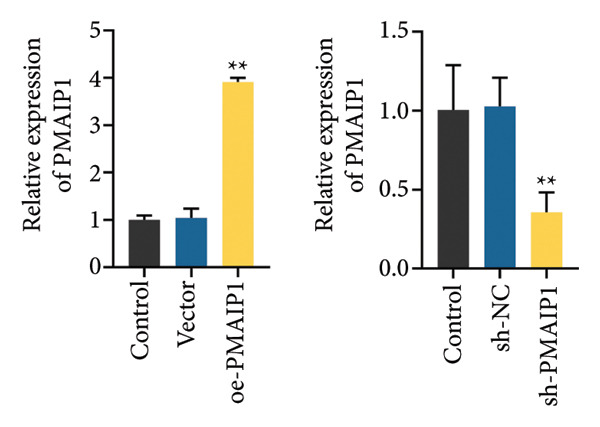
(c)

**Figure:**
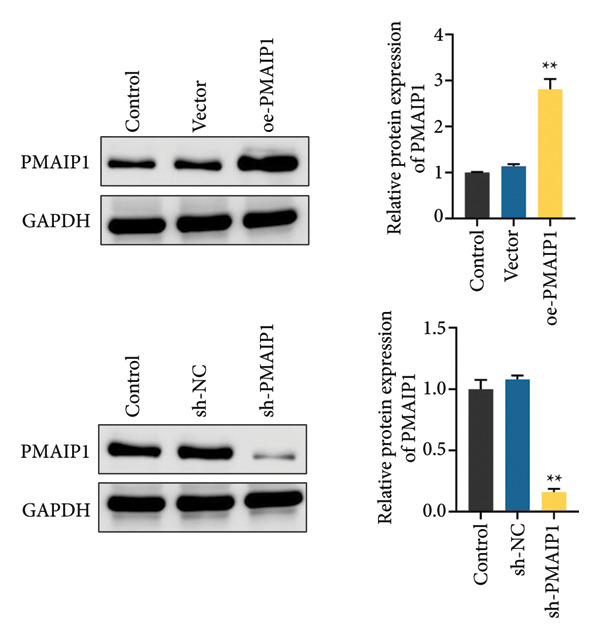
(d)

**Figure:**
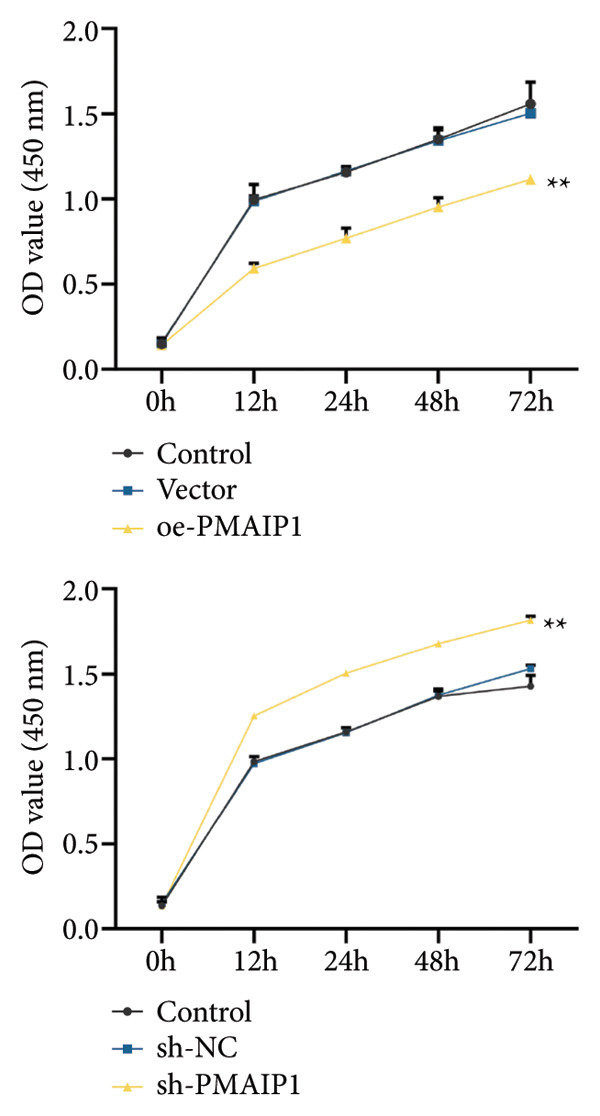
(e)

As illustrated in Figures 5(c), 5(d), 5(e), qRT‐PCR and western blot confirmed successful modulation of PMAIP1 expression. Cell viability, measured by MTT assay at baseline and up to 72 h post‐transfection, revealed that PMAIP1 overexpression significantly reduced proliferation in MDA‐MB‐468 cells in a time‐dependent manner, whereas PMAIP1 knockdown enhanced MDA‐MB‐231 cell viability (*p* < 0.05). These results align with the redox‐dependent apoptotic mechanism described above, supporting the notion that PMAIP1 inhibits TNBC cell growth through ROS accumulation and mitochondrial dysfunction‐induced apoptosis. Based on these molecular and phenotypic findings, we next integrated the experimental and bioinformatic evidence to further elucidate the potential mechanistic and clinical implications of PMAIP1 in TNBC pathophysiology.

## 4. Discussion

TNBC is the most aggressive subtype of breast cancer and lacks targeted therapeutic targets and effective treatments. This study systematically explored the role of PMAIP1 in TNBC through in vitro experiments and bioinformatics analysis, revealing its key regulatory role in apoptosis, DNA damage, and mitochondrial dysfunction, providing new insights into the treatment of TNBC.

Our study showed that the expression of PMAIP1 was significantly elevated in TNBC compared with normal breast tissue and cells. This is consistent with previous reports and supports the important role of PMAIP1 as a pro‐apoptotic gene in cancer [18, 20, 21]. Further experiments showed that upregulation of PMAIP1 could significantly inhibit the viability of TNBC cells and induce apoptosis. Through TUNEL detection and western blot analysis, it was found that PMAIP1 promotes the activation of the mitochondria‐mediated apoptosis pathway by upregulating Bax and inhibiting the expression of Bcl‐2 [22]. In contrast, knockdown of PMAIP1 enhanced cell viability and reduced apoptosis. These phenomena strongly suggest that PMAIP1 is a key regulator of apoptosis in TNBC, and its dysregulation may contribute to tumor progression [23].

It should be noted that although PMAIP1 was upregulated in TNBC tissues and in MDA‐MB‐231 cells, no significant difference was observed in MDA‐MB‐468 cells compared to MCF10A, reflecting the heterogeneity of TNBC subtypes. Importantly, the clinical observation that high PMAIP1 expression is associated with poorer prognosis in TNBC does not contradict its pro‐apoptotic role. Instead, elevated PMAIP1 likely represents a stress response to genomic instability, ROS accumulation, and p53 activation in aggressive tumors, while its apoptotic function may be counteracted by anti‐apoptotic factors such as Bcl‐2. Thus, high PMAIP1 expression should be regarded more as a marker of tumor aggressiveness than a straightforward tumor suppressor. In this context, our findings support a model in which PMAIP1 can promote mitochondrial apoptosis via the Bax/Bcl‐2 axis and ROS‐mediated dysfunction in vitro, but its clinical link to poor outcomes reflects context‐dependent regulation within the tumor microenvironment.

Additionally, this study identified a critical role for PMAIP1 in the DNA damage response. Overexpression of PMAIP1 significantly increased DNA damage levels, as evidenced by comet assay parameters and elevated expression of DNA damage markers such as γH2AX, p‐ATM, and p53. PMAIP1 may enhance DNA damage response signaling pathways, further undermining TNBC cell survival in concert with its pro‐apoptotic functions [23]. Moreover, PMAIP1‐induced oxidative stress and mitochondrial dysfunction were evident through increased ROS levels, decreased mitochondrial membrane potential, reduced ATP production, and compromised mtDNA integrity. These findings highlight the potential of PMAIP1 as a mediator of redox balance and mitochondrial dynamics in TNBC. The interplay between PMAIP1 and mitochondrial dysfunction offers new insights into the mechanisms underlying TNBC progression. Elevated ROS levels exacerbate mitochondrial damage, forming a feedback loop that promotes apoptosis [24–26]. By disrupting mitochondrial function, PMAIP1 overexpression sensitizes TNBC cells to oxidative stress, making it a promising target for redox‐based therapeutic strategies. In contrast, PMAIP1 knockdown alleviated oxidative stress and restored mitochondrial function, underscoring its regulatory role in maintaining cellular homeostasis.

Given the aggressive nature of TNBC and the lack of effective targeted therapies, the identification of PMAIP1 as a key regulator of apoptosis, DNA damage, and mitochondrial function suggests its potential as a novel therapeutic target. The ability of PMAIP1 to sensitize TNBC cells to oxidative stress and DNA damage highlights its relevance in redox‐based or genotoxic treatment strategies. Therapeutically, enhancing PMAIP1 expression or mimicking its downstream effects may improve the efficacy of existing chemotherapeutic agents, particularly those that rely on apoptotic priming or mitochondrial destabilization. Furthermore, combination approaches that activate PMAIP1 alongside DNA‐damaging agents (e.g., platinum‐based drugs or PARP inhibitors) may overcome therapeutic resistance and improve clinical outcomes. Future studies should explore druggable pathways upstream or downstream of PMAIP1 and assess whether its expression level could serve as a predictive biomarker for treatment response or patient stratification in TNBC.

Despite these findings revealing the critical role of PMAIP1 in TNBC, several limitations of this study must be acknowledged. First, all functional experiments were conducted in vitro, which may not fully capture the complexity of the tumor microenvironment or systemic factors influencing tumor progression [27, 28]. Future studies employing xenograft models or patient‐derived tumor xenografts will be essential to confirm these findings and assess therapeutic applicability. Second, while PMAIP1 was shown to regulate Bax/Bcl‐2 expression and elevate p53 levels, the mechanistic basis of these interactions remains unresolved. Further studies should perform co‐immunoprecipitation assays or utilize p53‐deficient TNBC models to determine whether PMAIP1 exerts its pro‐apoptotic effects through direct interactions or p53 dependency. Third, our analysis was limited to two commonly used TNBC cell lines. Although these models represent distinct molecular subtypes, expanding the range of TNBC cell lines or incorporating primary patient‐derived tumor cells would help generalize our findings and capture inter‐patient heterogeneity. Additionally, the specific molecular mechanisms underlying PMAIP1‐mediated mitochondrial and DNA damage pathways require further exploration, including potential interactions with other signaling pathways.

Furthermore, this study complements our previous findings by uncovering a novel ROS‐mitochondrial axis through which PMAIP1 mediates apoptosis and DNA damage in TNBC cells. These results provide mechanistic continuity with our earlier work while extending its implications toward oxidative stress‐based therapeutic strategies.

In summary, this study comprehensively elucidates the pivotal role of PMAIP1 in TNBC cell apoptosis, DNA damage, and mitochondrial dysfunction. By inhibiting tumor cell viability and disrupting cellular homeostasis through multiple mechanisms, PMAIP1 underscores its potential as a therapeutic target. At the same time, its elevated expression in clinical cohorts, based primarily on bulk transcriptomic analyses, should be interpreted as an indicator of tumor stress and aggressiveness rather than direct evidence of a simple tumor suppressor function. Future research into the mechanisms and regulatory strategies of PMAIP1 will lay the groundwork for novel therapeutic approaches for TNBC.

## 5. Conclusion

This study demonstrates that PMAIP1 regulates TNBC cell fate through a redox‐dependent mitochondrial pathway. Overexpression of PMAIP1 elevates intracellular ROS levels, disrupts mitochondrial function, and subsequently induces DNA damage and apoptosis, ultimately inhibiting cell viability. These findings uncover a novel ROS‐mitochondrial axis underlying the tumor‐suppressive effects of PMAIP1 and suggest that modulation of this pathway may offer a potential therapeutic strategy for TNBC.

## Disclosure

All authors read and approved the final manuscript.

## Conflicts of Interest

The authors declare no conflicts of interest.

## Author Contributions

Fangjian Shang and Lei Xu contributed to the study conception and design. Material preparation, data collection, and analysis were performed by Hongzhi Liu, Xin Dong, Huangfei Wu, Liping Yin, and Lijuan Yan. The first draft of the manuscript was written by Fangjian Shang, Lei Xu, Yixin Qi, and Liyan Zhao, and all authors commented on previous versions of the manuscript. Fangjian Shang and Lei Xu these authors contributed equally to this work.

## Funding

This study was supported by Hebei Provincial Colleges and Universities Youth Top‐Notch Talent Project Program (BJ2025056); Natural Science Foundation of Hebei Province (No. 22372409D); Hebei Provincial Department of Science and Technology Health Innovation Project (No. 20231063); Hebei Provincial Department of Science and Technology Health Innovation Project (No. 20240643); Hebei Medical University College Students’ Innovative Experimental Program Project (USIP2024107); Hebei Province Medical Research and Enterprise Joint Innovation Special Project Plan (LH20250146 and LH20250047); and Government‐funded Clinical Medicine Excellent Talent Training Project in 2026 (ZF2026055).

## Data Availability

The data used to support the findings of this study are available from the corresponding author upon request.
